# Nurses’ Adherence to Double‐Checking: A Systematic Review of Influencing Factors, Improvement Strategies, and Their Effectiveness

**DOI:** 10.1111/inr.70179

**Published:** 2026-04-15

**Authors:** Junyu Zhao, Hao Liu, Ao Shan, Yigong Peng, Calvin K. L. Or

**Affiliations:** ^1^ Department of Data and Systems Engineering The University of Hong Kong Hong Kong SAR China

**Keywords:** guideline adherence, human factors, medication errors, nurses, patient safety, quality improvement

## Abstract

**Aim:**

This systematic review aims to systematically (1) synthesize factors of nurses’ adherence to double‐checking, (2) identify and describe strategies used to improve adherence, and (3) assess the reported effectiveness of these strategies.

**Introduction:**

Medication administration errors are a major cause of adverse events. While two‐nurse double‐checking is widely mandated, nurses’ adherence is inconsistent and often not performed independently or is omitted when clinical demands compete. Existing reviews have not fully synthesized adherence determinants and improvement approaches across the work system.

**Methodology:**

Following PRISMA guidelines, we searched the CINAHL, PubMed, Embase, and Cochrane databases from inception to November 17, 2024. Peer‐reviewed studies examining adherence determinants and evaluating adherence‐improvement strategies were included. Determinants were mapped to a human factors engineering paradigm, and strategy findings were synthesized narratively.

**Results:**

Factors affecting adherence included interruptions, unavailability of a second nurse, high workload or time pressure, low perceived risk or necessity of double‐checking, and crowded medication rooms. Strategies used included written or visual reminders, education with feedback, and barcode‐supported auditing systems. Studies also reported that education paired with visual reminders and co‐sign tools, ongoing feedback with workflow integration, and protocolized barcode‐supported auditing improved adherence; however, evaluations were limited and inconsistent.

**Conclusions:**

Non‐adherence largely reflects constraints at all levels of the work system. Therefore, feasible and sustainable independent double‐checking requires the redesign of tasks, workflows, physical space and environments, resource allocation, and enabling mechanisms.

**Implications for nursing practice:**

Practical support should enable independent cognitive verification, timely access to a second checker, and safe communication during verification. Policies should define independent double‐checking, target requirements to higher‐risk situations, and pair mandates with staffing, workflow design, and usable technology that make adherence to double‐checking feasible and reviewable.

## 1 Introduction

### 1.1 Background

Medication administration is a high‐stakes task. As a key safety measure, double‐checking is widely promoted as a safety practice and is commonly mandated for selected high‐risk medications (Hewitt et al. 2016; Koyama et al. 2020). In this review, double‐checking refers to a procedure in which two nurses verify key patient and medication details before administration (Ek et al. 2022). The purpose is to catch potential errors that a single nurse might miss, thereby acting as a safeguard in the medication administration process (van Stralen et al. 2024). Importantly, double‐checking is not always operationalized as a uniform intervention, since routine checking behaviors can range from truly independent verification to more collaborative or “primed” forms of confirmation, so documented completion does not necessarily equate to high‐fidelity (independent and cognitively active) checking (Hawkins and Morse 2022; Milic et al. 2023).

**What is already known**:

- Double‐checking by two nurses is a widely promoted safety practice intended to prevent medication administration errors.
- Nurse adherence to double‐checking protocols is often inconsistent in real‐world practice.

**What this paper adds**:

- Key barriers to nurses’ adherence to double‐checking include frequent interruptions, lack of a second nurse, low perceived risk or necessity of double‐checking, high workloads, and crowded work environments.
- Various improvement strategies have been shown to increase nurses’ double‐checking adherence, including written or visual reminders, educational sessions, and barcode‐based tracking.
- Specific recommendations are provided for nursing practice, policy, and research to support sustained improvements in double‐checking adherence.

Despite its theoretical benefit, implementing double‐checking in real‐world practice presents challenges. The process requires two nurses and extra time, which can disrupt workflow in busy settings and strain nursing resources (Shan et al. 2023). As a result, nurses do not always adhere to double‐checking, especially under high workload or urgent conditions (Alsulami et al. 2014b; Vaismoradi et al. 2020). Reported adherence to mandated double‐checking varies widely. In observational studies, nurses performed the required double‐checking in roughly 50% of dose administrations to almost 100%, depending on the context (Koyama et al. 2020). Such variability suggests that double‐checking is often informally triaged: for instance, a recent qualitative study found that time pressure was a key barrier, with nurses consciously deciding to skip double‐checking for low‐risk situations or when they felt confident in their own and colleagues’ accuracy (van Stralen et al. 2024). This gap between policy, or work‐as‐imagined, and bedside practice, or work‐as‐done, may reflect rational adaptation to other demands, consistent with an efficiency–thoroughness trade‐off in time‐pressured clinical work. Other practical issues include the risk of confirmatory bias if the second check is not truly independent and the tendency for double‐checking to become superficial if rushed (Koyama et al. 2020; Vaismoradi et al. 2020). In addition, double‐checking may unintentionally shift responsibility between the two checkers, potentially reducing individual attention when roles, expectations, or independence are unclear. Consequently, although double‐checking is intended to improve patient safety, its actual effectiveness in reducing medication administration errors may be undermined when adherence is inconsistent (Koyama et al. 2020). This tension between protocol and practice highlights the need to clarify when, why, and how nurses adhere to high‐fidelity double‐checking in everyday medication administration (Schroers et al. 2025; Shan et al. 2023).

Existing literature on medication double‐checking reveals important gaps and inconsistent findings. High‐quality evidence that double‐checking reliably improves patient outcomes remains lacking (Konwinski et al. 2024). A later systematic review reached a similar conclusion, reporting insufficient evidence that double‐checking, compared with single‐checking, reduces medication administration errors or related harm; among the three good‐quality studies it identified, only one showed a significant association, one showed no association, and one reported adherence rates only (Koyama et al. 2020). A more recent analysis also revealed a similar conclusion, with a 2020 review reporting insufficient evidence that double‐checking (vs. single‐checking) is associated with lower medication administration error rates or reduced harm (Koyama et al. 2020). Among the limited rigorous studies, findings have been mixed: one study noted a significant reduction in administration errors with double‐checking, whereas others found no significant difference (Cioccari et al. 2024; Vaismoradi et al. 2020). Also, few randomized controlled trials have been conducted on this topic, and many publications are descriptive or qualitative (Koyama et al. 2020; Vaismoradi et al. 2020). However, interpreting outcome findings is challenging because “double‐checking” is often inconsistently defined and measured across studies, and adherence is frequently operationalized as documentation of a second check rather than the independence and quality of the double‐checking process (Ek et al. 2022; Milic et al. 2023). Taken together, these findings suggest that the effectiveness of double‐checking may depend heavily on how it is implemented in practice, making adherence a critical issue to understand.

Moreover, despite its importance, it is currently less clear what factors influence nurses’ adherence to double‐checking and how to improve it. While some studies document that adherence is suboptimal, the reasons for non‐adherence have not been comprehensively reviewed. Qualitative evidence suggests several contributing factors, such as stress, high workload, complex medication processes, similarities in drug names, and inadequate training. Nurses may also treat double‐checking as an automatic, routine task, leading to reduced vigilance (McMullan et al. 2023). They might diffuse responsibility between the two checkers, and junior nurses might hesitate to question a senior's preparation (Koyama et al. 2020). Environmental and organizational factors, such as inadequate staffing, high workload, distractions, or unclear hospital policies, are also frequently cited as potential barriers in individual studies. Moreover, tool and technology conditions may shape how checks are performed in practice and may also influence adherence to protocol when systems are poorly aligned with protocol constraints. Without a consolidated synthesis of determinants, it is difficult to design interventions that are both feasible and responsive to real‐world conditions.

Likewise, research on strategies to improve double‐checking adherence is limited. Approaches such as training sessions emphasizing independent verification and the addition of checklists or co‐signature reminders in electronic medication administration records have been trialed. However, evaluations remain sparse, and results are inconsistent (Ghezaywi et al. 2024; Raghavan et al. 2022). Accordingly, a comprehensive synthesis is required to summarize the factors influencing adherence, the strategies implemented to improve it, and whether these strategies yield meaningful effects.

This review is framed using a human factors engineering paradigm (Karsh et al. 2006) to analyze the factors associated with nurses' adherence to double‐checking. Human factors engineering is a discipline that considers human strengths and limitations in the design of work systems involving tasks, tools, technology, physical environments, and organizations, with the goal of ensuring safety and efficiency (Karsh et al. 2006). In practice, a human factors approach examines how various system elements interact and influence performance. Applying a human factors engineering perspective is pertinent to medication double‐checking because this practice lies at the intersection of human behavior and work system design. By understanding double‐checking as part of a larger work system, we can categorize barriers and facilitators into six domains: person factors, task factors, tool/technology factors, physical environment factors, organizational factors, and external factors. The human factors engineering paradigm provides a structured framework to ensure that none of these domains are overlooked when analyzing nurses’ adherence to double‐checking. Importantly, this systematic lens also supports attention to implementation fidelity, not only whether double‐checking occurs, but also how work system conditions shape the independence and quality of checking in real practice.

The rationale for using human factors engineering in this context is its proven utility in improving patient safety by identifying system‐level contributors to human error. High‐risk industries like aviation and nuclear power have long employed human factors engineering principles, e.g., to develop standardized checklists and redundancies to minimize errors (Moray and Huey 1988; Salas and Maurino 2010). In healthcare, a human factors engineering approach aligns with the understanding that errors are seldom simply “individual failures”; rather, they often stem from poorly designed processes or system pressures (Carayon et al. 2014; Reason 2000; Scanlon and Karsh 2010). By employing a human factors engineering paradigm, this review organizes findings about double‐checking adherence in a way that highlights interactions between nurses and the systems in which they work (Li and Carayon 2021; Melles et al. 2021). Therefore, this review synthesizes associated factors from a holistic, systems‐oriented perspective to inform feasible, effective strategies for strengthening double‐checking adherence in complex healthcare settings. Guided by this system‐oriented framework, we conducted a systematic review to integrate evidence on determinants of adherence and the strategies used to improve it.

This systematic review aims to achieve the following objectives:

1. Systematically synthesize current knowledge on the factors influencing nurses’ adherence to double‐checking during medication administration.
2. Identify strategies that were used to improve nurses’ adherence to double‐checking.
3. Assess the reported effectiveness of these strategies in improving nurses’ adherence to double‐checking.

The review seeks to provide a comprehensive and evidence‐informed understanding of why double‐checking practices may falter and what can be done to strengthen adherence to double‐checking.

## 2 Methodology

This review was reported in accordance with the PRISMA 2020 statement for systematic reviews (Page et al. 2021).

### 2.1 Search Strategy

We searched CINAHL, PubMed, Embase, and Cochrane databases from inception to November 17, 2024, for studies on nursing and double‐checking. The search terms included variations of “nurse” (to encompass all nursing professionals) and terms related to “double‐checking” (such as “double check,” “double‐checking,” and synonyms for verification by a second person). Full search strings are in the Appendix.

### 2.2 Inclusion Criteria

Studies were included in this review if they (1) examined nurses, defined as licensed/regulated nursing staff, including registered nurses and enrolled (second‐level) nurses or equivalent titles, (2) assessed factors influencing nurses’ adherence to double‐checking or evaluated interventions/strategies aimed at improving nurses’ adherence to double‐checking, and (3) were published in peer‐reviewed journals in English.

### 2.3 Study Selection

After removing duplicates, two reviewers (JZ and YP) independently screened titles and abstracts against inclusion criteria. Potentially relevant studies underwent full‐text assessment. Reference lists of included studies were hand‐searched for additional relevant articles. Interrater agreement during screening was substantial (Cohen's κ = 0.71). Disagreements were resolved by discussion and consensus.

### 2.4 Data Extraction

Two reviewers independently extracted data from included studies using a pre‐developed standardized form that defined all required fields and provided instructions for consistency. For each study, they extracted the specific factors or strategies examined, adherence‐related outcomes reported, key study characteristics (author, year of publication, location/setting, duration, sample size, and participant demographics), and effectiveness outcomes of the strategies. Discrepancies were resolved through discussion until consensus was reached.

### 2.5 Outcome Measures

This review examined the following outcomes: factors influencing nurses’ adherence to double‐checking protocols, strategies aimed at improving nurses’ adherence to double‐checking, and their reported effectiveness. For strategy‐focused studies, we extracted the strategy components and implementation characteristics, then synthesized the reported evaluation outcomes. Studies describing adherence‐improvement strategies without a formal effectiveness evaluation were also eligible for inclusion and were summarized descriptively.

### 2.6 Quality Appraisals

The methodological quality of each included study was assessed independently by two reviewers. For qualitative studies, we used the Critical Appraisal Skills Program (CASP 2018). For descriptive comparison across studies, we converted the first nine CASP questions into a review‐specific summary score from 0 to 9; the tenth question on the value of the research was considered narratively. For quantitative studies, the relevant JBI checklist was applied according to study design (Barker et al. 2024). Because JBI checklists vary in the number of items across designs, checklist responses were converted into a review‐specific standardized score from 0 to 10 for descriptive comparison across studies. Any discrepancies in ratings between the two reviewers were discussed and resolved by consensus.

### 2.7 Human Factors Engineering Paradigm

This review adopted the human factors engineering paradigm (Karsh et al. 2006) to guide our analysis of the factors affecting nurses' adherence. The paradigm allowed us to consider not only individual nurse behaviors, but also how system‐level factors could facilitate or hinder adherence to double‐checking. The paradigm emphasizes proactive system design and optimization, aiming to enhance overall work processes and reduce opportunities for errors. Using the human factors engineering lens, we categorized each identified factor into one of the six interrelated domains:

- **Patient and Healthcare Provider Factors**: Individual characteristics of patients and healthcare providers, such as health status, age, experience, needs, training, and knowledge, can influence adherence behaviors.
- **Task Factors**: These encompass workflow, work complexity, time pressure, procedural demands, and job control. High task complexity or time constraints can reduce adherence, especially in high‐risk clinical settings.
- **Technology and Tool Factors**: This domain assesses the availability, design (e.g., reliability, functionality, and usability), and integration (e.g., compatibility with other technologies and elements of other domains) of technologies and tools.
- **Physical Environment Factors**: Factors related to the physical workspace, such as lighting, noise, temperature, and spatial layout, can affect task performance.
- **Organizational Factors**: Organizational elements, including management structure, safety culture/climate, leadership practices, hierarchical dynamics, and policy enforcement, can shape adherence.
- **External Factors**: Broader elements, such as legislation, extra‐organizational standards, economic constraints, and industry social influence, can affect adherence by shaping organizational elements.

### 2.8 Data Analysis

Two reviewers (JZ and YP) independently categorized each identified factor into the appropriate human factors engineering domain, resolving disagreements through discussion and consensus. Due to heterogeneity of adherence‐improvement strategies, quantitative synthesis was not feasible; narrative synthesis was therefore used. Specifically, the reviewers summarized the implemented strategies and their reported effects on double‐checking adherence.

## 3 Results

### 3.1 Study Selection

**FIGURE 1:**
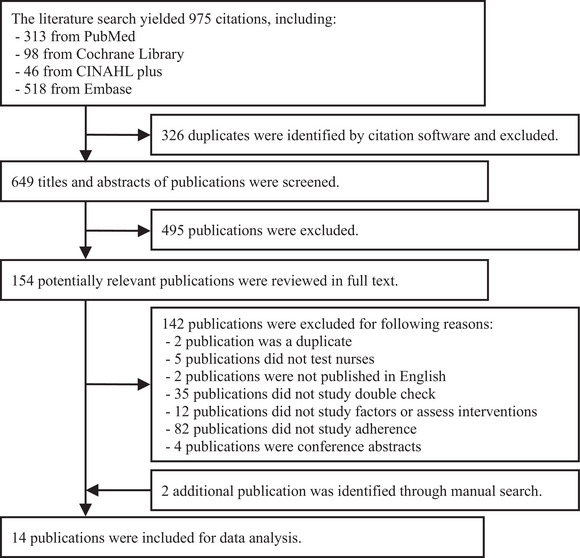
Flow diagram of the study selection process.

Figure 1 presents the study selection process, following the PRISMA reporting guidelines. While a meta‐analysis was not feasible, the process yielded 14 eligible publications.

### 3.2 Characteristics of Included Studies

Table 1 summarizes the characteristics of the 14 studies included in the review.

**TABLE 1:** Characteristics of studies that were included in the review.

Characteristics	Number of studies involved (%)
Year of publication	
2005–2009	3 (21%) (Manias et al. 2005; Hospodar 2007; Raja Lope et al. 2009)
2010–2014	4 (29%) (Dickinson et al. 2010; Gill et al. 2012; Alsulami et al. 2014a, 2014b)
2015–2019	4 (29%) (Fang et al. 2016; Subramanyam et al. 2016; Soltanian et al. 2018; Schutijser et al. 2019)
2020–2024	3 (21%) (Nakamura et al. 2021; Westbrook et al. 2021; van Stralen et al. 2024)
Study location	
America	2 (14%) (Hospodar 2007; Subramanyam et al. 2016)
Europe	4 (29%) (Alsulami et al. 2014a, 2014b; Schutijser et al. 2019; van Stralen et al. 2024)
Asia	4 (29%) (Raja Lope et al. 2009; Fang et al. 2016; Nakamura et al. 2021; Soltanian et al. 2018)
Oceania	4 (29%) (Manias et al. 2005; Dickinson et al. 2010; Gill et al. 2012; Westbrook et al. 2021)
Study examined factors affecting adherence to double‐checking	10 (71%) (Manias et al. 2005; Dickinson et al. 2010; Gill et al. 2012; Alsulami et al. 2014a, 2014b; Soltanian et al. 2018; Schutijser et al. 2019; Nakamura et al. 2021; Westbrook et al. 2021; van Stralen et al. 2024)
Study examined double‐checking adherence‐improvement strategies	4 (29%) (Hospodar 2007; Raja Lope et al. 2009; Fang et al. 2016; Subramanyam et al. 2016)

### 3.3 Quality Appraisal

The six qualitative studies achieved an average quality appraisal score of 7 out of 9 (range 6–8; see Table 2). Common concerns related to participant recruitment strategies and insufficient detail in data analysis. The eight non‐randomized trials achieved an average quality appraisal score of 8.1 out of 10 (range 6–10; see Table 3). Studies evaluating adherence‐improvement strategies often omitted nurse demographic details, while those examining adherence factors often provided limited information about study sites and settings.

**TABLE 2:** Quality appraisal results of qualitative studies.

	Q1. Was there a clear statement of the aims of the research?	Q2. Is a qualitative methodology appropriate?	Q3. Was the research design appropriate to address the aims of the research?	Q4. Was the recruitment strategy appropriate to the aims of the research?	Q5. Were the data collected in a way that addressed the research issue?	Q6. Has the relationship between researcher and participants been adequately considered?	Q7. Have ethical issues been taken into consideration?	Q8. Was the data analysis sufficiently rigorous?	Q9. Is there a clear statement of findings?	Overall score	Q10. How valuable is the research?
Manias et al. (2005)	Yes	Yes	Yes	Yes	No	Yes	No	Yes	No	6	The study provides a thorough analysis of nurses’ medication administration process.
Dickinson et al. (2010)	Yes	Yes	Yes	No	No	Yes	Yes	No	Yes	6	The study exhibits direct relevance to the subject, contributing valuable insights.
Gill et al. (2012)	Yes	Yes	Yes	Yes	No	Yes	No	Yes	Yes	8	The study is well conducted and is valuable in the context.
Soltanian et al. (2018)	Yes	Yes	Yes	No	Yes	Yes	Yes	No	Yes	7	Though not in direct alignment with the review's topic, this study's outcomes encompass factors associated with nurses’ adherence.
Schutijser et al. (2019)	Yes	Yes	Yes	Yes	No	Yes	No	Yes	Yes	8	The study is well conducted and is valuable in the context.
van Stralen et al. (2024)	Yes	Yes	No	Yes	Yes	Yes	Yes	No	Yes	7	The study analyzed nurses’ behavior using a new framework and provided insights.

**TABLE 3:** Quality appraisal of included non‐randomized trials.

	Q1. Were there clear criteria for inclusion in the case series?	Q2. Was the condition measured in a standard, reliable way for all participants included in the case series?	Q3. Were valid methods used for identification of the condition for all participants included in the case series?	Q4. Did the case series have consecutive inclusion of participants?	Q5. Did the case series have complete inclusion of participants?	Q6. Was there clear reporting of the demographics of the participants in the study?	Q7. Was there clear reporting of clinical information of the participants?	Q8. Were the outcomes or follow‐up results of cases clearly reported?	Q9. Was there clear reporting of the presenting sites/clinics?	Q10. Was statistical analysis appropriate?	Overall score
Hospodar (2007)	Yes	No	Yes	Yes	Yes	Yes	No	Yes	Yes	Yes	8
Raja Lope et al. (2009)	No	Yes	Yes	Yes	Yes	No	Yes	No	Yes	Yes	7
Alsulami et al. (2014a)	Yes	Yes	Yes	No	Yes	Yes	Yes	Yes	Yes	Yes	9
Alsulami et al. (2014b)	Yes	Yes	Yes	Yes	Yes	Yes	Yes	Yes	Yes	Yes	10
Fang et al. (2016)	Yes	No	Yes	Yes	Yes	No	Yes	Yes	Yes	Yes	8
Subramanyam et al. (2016)	Yes	Yes	No	No	Yes	No	Yes	Yes	No	Yes	6
Nakamura et al. (2021)	Yes	Yes	Yes	Yes	No	Yes	Yes	Yes	No	Yes	8
Westbrook et al. (2021)	Yes	Yes	Yes	No	Yes	Yes	Yes	Yes	Yes	Yes	9

### 3.4 Factors Affecting Nurses’ Adherence to Double‐checking

Table 4 summarizes factors influencing nurses’ adherence to double‐checking from the included studies and key study characteristics. Table 5 categorizes these factors using the human factors engineering paradigm, showing the number of studies per factor. Details of the factors are presented below, with study types indicated in brackets.

**TABLE 4:** Factors influencing double‐checking adherence and their study details.

Author (year), location	Study type, duration, settings	Sample size (*N*) and demographics	Study findings	Characteristics of double‐checking
Manias et al. (2005), Australia	Descriptive qualitative. Sept–Oct 2002. Metropolitan teaching hospital (medical, surgical, and various specialty wards).	*N* = 12. Age: 21–45 years (mean = 25.5, SD = 6.7). Gender: One (8.3%) male nurse and 11 (91.7%) female nurses. Work experience: All participants were eight to 10 months into their 12‐month graduate nurse program.	Graduate nurses double‐checked the preparation of designated medication in 97% of situations, while they double‐checked medications at patients’ bedside in 80% of situations.	Mandatory 2‐Person Check: Required for specific meds (e.g., parenteral/opioids). Involved a grad nurse and another RN (protocol‐driven). Occurs during preparation and administration. Independence not specified.
Dickinson et al. (2010), New Zealand	Descriptive qualitative. Duration not reported. Major children's hospital inpatient areas (medical, surgical, ICU, oncology, cardiac).	*N* = 19. Age and gender are not reported. Six Level 1/2 nurses (newly registered or confident in uncomplicated situations); seven Level 3/4 nurses (confident in complex situations or specialists); six senior nurses (management or senior clinical leadership roles).	Environmental factors that reduced adherence include overcrowding in the medication rooms, interruptions during preparation, competing priorities and high workload demands, and difficulties accessing pediatric medication resources to guide medication administration. Attitudinal factors that reduced adherence include complacency toward double‐checking, approachability, trustworthiness, and availability of a second nurse, and junior nurses’ reluctance to challenge senior nurses.	Recommended 2‐Person Check: Promoted as best practice (IDC) for dose calculation, prep, and admin. Implementation varied due to a lack of clarity on “independent” meaning.
Gill et al. (2012), Australia	Qualitative study. Duration not reported. Australian tertiary children's hospital (surgical wards and pediatric ICU).	*N* = 24. Age and gender are not reported. Work experience: Four graduate registered nurses and 20 registered nurses with more than one year of experience.	Patient factors (younger age, less familiarity with patients) and environmental factors (higher perceived medication danger, better protocol‐compliant ward culture, low confidence in colleagues) increased adherence to double‐checking. Night shifts, busy periods, and graduate nurses’ time pressure (due to task unfamiliarity and longer durations) reduced adherence. Senior nurses skipping double‐checking further negatively impacted graduate nurses’ adherence.	Mixed Mandatory Protocol: 2‐person check mandatory for Schedule 8/controlled drugs; single nurse for others. Verified details at bedside. Independence described in protocol but not explicit.
Alsulami et al. (2014a), United Kingdom	Cross‐sectional. July–Aug 2012. Four inpatient areas in a children's hospital (medical, surgical, PICU, NICU).	*N* = 48. Age: 21–30 (35.4%, *n* = 17), 31–40 (14.6%, *n* = 7), 41–50 (20.8%, *n* = 10), >51 (29.2%, *n* = 14). Gender: Two male nurses (4.2%) and 46 female nurses (95.8%). Work experience: <1 year (2.1%, *n* = 1), 1–2 years (4.2%, *n* = 2), 2–5 years (12.5%, *n* = 6), 5–10 years (22.9%, *n* = 11), >10 years (58.3%, *n* = 28).	A majority (83.3%, *n* = 40) considered pediatric nurse shortages and high workload as barriers to double‐checking adherence, while 85.4% (*n* = 41) agreed that disturbances and interruptions are barriers. Nearly two‐thirds (64.6%, *n* = 31) reported that the unavailability of a second nurse reduced adherence. Although most (93.8%, *n* = 45) observed no difference in adherence between weekdays and weekends, 31.3% (*n* = 15) reported higher adherence during night shifts due to fewer interruptions.	Mandatory 2‐Person Process: Required by policy for the whole process (identifying need for administration). Involved two registered pediatric nurses. Independence details not available due to limited knowledge of policy.
Alsulami et al. (2014b), United Kingdom	Observational. Apr–July 2012. Children's hospital inpatient wards (medical, surgical, paediatric ICU, neonatal ICU).	*N* was not reported. Age, gender, and work experience were not reported.	Dose calculations had the lowest adherence rate (30%), followed by flush‐syringe label checks (67%), intravenous bolus‐rate checks (71%), and administration to patients (83%). The remaining 11 steps all had adherence rates above 90%. Double‐checking adherence rates were significantly higher on weekends compared with weekdays.	Mandatory 2‐Person Check: Policy required double‐checking for all meds during prep and admin. Independent dose calculation was expected (but with low adherence).
Soltanian et al. (2018), Iran	Qualitative study. Duration not reported. Teaching hospital, pediatric wards.	*N* = 15. Age: mean 31.7 years. Gender is not reported. Work experience: mean 7.8 years.	Nurses are more likely to conduct double‐checking for high‐alert drugs. Nurses with more loyalty to duty prioritize safe medication administration as part of their professional responsibility and have higher adherence to double‐checking.	Mandatory for High‐Alert Drugs: 2‐person check before injection of high‐alert drugs. One‐person check for others.
Schutijser et al. (2019), Netherlands	Qualitative study. Feb–July 2018. Internal medicine and surgery wards across two Dutch hospitals.	*N* = 27. Age is not reported. Gender: Five (18.5%) male nurses and 22 (81.5%) female nurses. Work experience: median was 8 years.	Facilitators include sufficient understanding of double‐checking's importance, a critical, collegial, and feedback‐oriented team culture, electronic health records with medication administration records and barcode systems, availability of trainees, and pharmacy‐prepared medications.	Protocol Independent 2‐Person Check: Mandatory for injectables. Protocol requires independent calc/check; practice often involves “priming.”
			Barriers include skepticism about the effectiveness of double‐checking, increased workload, computer system failures, staff shortages, lack of time, frequent interruptions, and an unclear pharmacy medication preparation process.	
Nakamura et al. (2021), Japan	Cross‐sectional study. 2018. Tertiary‐care teaching hospital (inpatient wards, ORs, outpatient clinics).	*N* = 1015. Age and gender are not reported. Work experience: ≤1 y (15%, *n* = 155), 2–10 y (64%, *n* = 645), ≥11 y (21%, *n* = 206).	The rate of asking another person to accompany them at the bedside and recognizing the necessity of two‐person signing of the blood sample label was significantly lower among nurses with ≤1 year of experience and no experience in transfusion practice.	Mandatory 2‐Person Check: Standard visual/verbal check + barcode ID. Required for blood sample collection and transfusion administration.
Westbrook et al. (2021), Australia	Observational study. Apr–Sept 2016. Tertiary paediatric hospital (nine medical and surgical wards).	*N* = 5,140 dose administrations (3,563 with mandatory double‐checking and 1,577 with optional double‐checking). Age, gender, and work experience are not applicable, as *N* refers to dose administrations, not to participants.	For mandatory double‐checking, 1.0% are independently double‐checked, 92.5% are primed double‐checked, and 6.5% received incomplete or no double‐checking. Factors associated with higher mandated double‐checking adherence include working on the orthopedics ward, registered nurses, weekend, and using a paper‐based system. For optional double‐checking, factors associated with higher adherence include less than 2 years of experience, not multitasking, using a paper‐based system, administering medications after 6 pm, and administering non‐oral medications.	Mandatory & Optional Independent Checks: Policy requires an independent 2‐person check for most meds; optional for others. In practice, independent checks were rare; most were “primed.”
van Stralen et al. (2024), Netherlands	Qualitative study. June 2020–June 2021. Multiple Dutch hospitals (10 adult wards: internal medicine, surgery, ICU).	*N* = 77. Age is not reported. Gender: 70 (91%) female nurses. Work experience: mean 13 years.	The following factors are associated with higher adherence: (1) the presence of a second nurse; (2) lower levels of knowledge, experience, and confidence; (3) classification of medications as high‐risk; (4) a higher perceived risk linked to the medication and patient's condition; (5) complexity of the administration process; (6) higher difficulty in dosage calculations; (7) higher levels of crowding in the ward.	Guideline Independent 2‐Person Check: Mandatory for high‐risk meds. Intended as an independent bedside check; practice varied (priming, retrospective check, or skipped based on risk assessment).

**TABLE 5:** Categorized factors based on the human factors engineering paradigm.

Factor types	Specific factors and the reported direction of association with double‐checking adherence
Patient and healthcare professionals factors	Nurse–patient familiarity: higher familiarity → lower adherence (Gill et al. 2012); Patient age (pediatric): younger age → higher adherence (Gill et al. 2012); Patient clinical complexity: higher complexity → higher adherence (van Stralen et al. 2024); Nurse's perceived necessity and effectiveness of double‐checking: higher perceived necessity/effectiveness → higher adherence; doubts/skepticism → lower adherence (Schutijser et al. 2019); Familiarity with the medication: greater familiarity → lower adherence (van Stralen et al. 2024); Confidence in other nurses’ checking: lower confidence → higher adherence (Gill et al. 2012); Qualification (registered vs. enrolled): registered nurses → higher adherence (Westbrook et al. 2021); Clinical experience: mixed (less experience → higher adherence (Westbrook et al. 2021; van Stralen et al. 2024); ≤1 year with no transfusion practice → lower adherence for bedside checking (Nakamura et al. 2021)).
Task factors	Variation by specific step: dose calculation → lower adherence, and (for IV meds) IV bolus‐rate verification → lower adherence, flush‐syringe labeling → lower adherence; bedside administration check → lower adherence relative to other steps (Dickinson et al. 2010; Alsulami et al. 2014a, 2014b); Perceived medication risk level: higher perceived risk → higher adherence (van Stralen et al. 2024; Gill et al. 2012; Soltanian et al. 2018); Double‐checking location: medication room vs. bedside: medication room → higher adherence (Manias et al. 2005); Multitasking status: multitasking → lower adherence; not multitasking → higher adherence (Westbrook et al. 2021); Medication administration route: non‐oral routes during administration → higher adherence (van Stralen et al. 2024; Westbrook et al. 2021); Calculation difficulty and confidence: difficult calculations/lack of confidence → higher adherence (van Stralen et al. 2024); Workload and staffing pressure: higher workload/ staff shortages → lower adherence (van Stralen et al. 2024; Dickinson et al. 2010; Alsulami et al. 2014a; Schutijser et al. 2019); Shift timing and time of administration: mixed; night shift perceived easier due to fewer interruptions (Alsulami et al. 2014a); after 18:00 associated with higher optional double‐checking (Westbrook et al. 2021);
Technology and tool factors	Medication chart format (paper vs. eMM): paper‐based charts → higher adherence (Westbrook et al. 2021); EHR and barcode systems: perceived facilitator → higher adherence (Schutijser et al. 2019).
Environmental factors	Interruptions during administration: more interruptions → lower adherence (Dickinson et al. 2010; Alsulami et al. 2014a; Schutijser et al. 2019); Overcrowding in medication areas and wards: overcrowding → lower adherence (Dickinson et al. 2010; van Stralen et al. 2024); Clinical emergent situations: emergent situations → lower adherence (Nakamura et al. 2021).
Organizational factors	Second‐checker unavailability/“no attendance” → lower adherence (van Stralen et al. 2024; Alsulami et al. 2014a; Nakamura et al. 2021); Increased staffing capacity (including trainees) → higher adherence (Schutijser et al. 2019); Low approachability/trustworthiness of available second checkers → lower adherence (Dickinson et al. 2010); Orthopedics ward (vs reference ward) → higher adherence (Westbrook et al. 2021); Ward culture of omitting checks/normalization of non‐checking → lower adherence (Gill et al. 2012); Collegial, critical, feedback‐oriented team culture → higher adherence (Schutijser et al. 2019); Difficulty accessing pediatric medication resources → lower adherence (Dickinson et al. 2010); Use of informal alternatives to formal ID verification → lower adherence (Gill et al. 2012); Low acceptability/perceived impracticality of protocol → lower adherence (Gill et al. 2012); Pharmacy‐prepared injectable medications → higher adherence (Schutijser et al. 2019); Lack of overview of pharmacy preparation process → lower adherence (Schutijser et al. 2019)
External environment factors	Not applicable.

#### 3.4.1 Patient and Healthcare Professionals Factors

##### 3.4.1.1 Nurse Familiarity With Patients

Registered pediatric nurses reported widespread non‐adherence to the patient identification step of double‐checking, particularly when they were familiar with the patient (focus group) (Gill et al. 2012).

##### 3.4.1.2 Patient Age (Pediatric)

Pediatric nurses reported greater adherence to double‐checking when administering medications to younger pediatric patients, attributing this to their perception of higher medication error risk in this age group (focus group) (Gill et al. 2012).

##### 3.4.1.3 Patient Clinical Complexity

Nurses reported conducting an informal risk assessment to determine the necessity for double‐checking. They reported a greater inclination to double‐check when patients presented with higher complexity, for example, those who received palliative care or those with multimorbidity and associated multiple medications (qualitative) (van Stralen et al. 2024).

##### 3.4.1.4 Nurses’ Perceived Necessity and Effectiveness of Double‐Checking

Nurses’ adherence was influenced by their beliefs about the necessity and effectiveness of double‐checking. Most viewed it as essential and error‐reducing, while a minority questioned its value for medication safety, which acted as a barrier to adherence (semi‐structured group interview) (Schutijser et al. 2019).

##### 3.4.1.5 Nurses’ Familiarity With the Administered Medication

Nurses reported that greater familiarity with the medication (e.g., frequent or recent administration) reduced their likelihood of requesting a second nurse for a double‐check, which reduced adherence (qualitative) (van Stralen et al. 2024).

##### 3.4.1.6 Nurses’ Confidence in Other Nurses’ Checking

Nurses were more likely to conduct double‐checking when they had less confidence in the other nurses involved in the checking process (focus group) (Gill et al. 2012).

##### 3.4.1.7 Nurse Qualification (Registered Nurses vs. Enrolled Nurses)

Among pediatric inpatients, registered nurses showed higher adherence to mandated double‐checking than enrolled nurses (OR = 1.58 (1.07–2.32)) (observational) (Westbrook et al. 2021).

##### 3.4.1.8 Nurses’ Clinical Experience Level

Clinical experience influenced adherence, with effects varying by task and context. Nurses with <2 years of experience were more likely to perform optional double‐checking (observational) (Westbrook et al. 2021). Similarly, less experienced nurses reported a greater inclination to request a double‐check before medication preparation (qualitative) (van Stralen et al. 2024). In contrast, a study of transfusion‐related bedside checks showed that nurses with ≤1 year of experience and no prior transfusion practice were less likely to request a second nurse, thereby reducing adherence (cross‐sectional) (Nakamura et al. 2021).

#### 3.4.2 Task Factors

##### 3.4.2.1 Variation by Specific Step

Adherence varied across steps. Adherence reached or exceeded 90% for 11 of the 15 observed steps (11 standard medication administration steps plus 4 additional intravenous‐specific steps). However, independent double‐checking of dose calculations occurred in fewer than one‐third of cases. For intravenous medications, adherence was particularly low for verifying IV bolus administration rates and labeling flush syringes. Double‐checking at the point of medication administration to patients also fell below 90% (observational) (Alsulami et al. 2014b).

##### 3.4.2.2 Handling Medications Viewed as High Risk

Nurses reported more consistent double‐checking when handling controlled drugs, medications viewed as particularly “dangerous,” or blood products (focus group) (Gill et al. 2012). Pediatric nurses described carefully double‐checking high‐alert medications, such as digoxin, insulin, potassium chloride, diazepam, barbiturates, heparin, and magnesium sulfate, to prevent medication errors (qualitative) (Soltanian et al. 2018). Nurses also weighed medication‐related risk, including the potential consequences and adverse effects of an error, when deciding whether a double‐check was necessary (qualitative) (van Stralen et al. 2024).

##### 3.4.2.3 Double‐Checking Location: Medication Room vs. Bedside

Graduate nurses demonstrated higher adherence during medication preparation in the medication room than at the bedside (97% vs. 80%) (qualitative) (Manias et al. 2005). This was attributed to greater convenience in the medication room, where nurses were already gathered, while bedside checking required an extra step and felt more time‐consuming (Manias et al. 2005).

##### 3.4.2.4 Multitasking Status During Administration

Adherence to optional double‐checking was higher when nurses were not multitasking during medication administration (observational) (Westbrook et al. 2021).

##### 3.4.2.5 Medication Administration Route

Nurses were more likely to perform optional double‐checking when medications were administered via non‐oral routes (e.g., inhalation, IV infusion, or IV injection) compared with oral administration (observational) (Westbrook et al. 2021). Nurses reported that greater procedural complexity during administration (e.g., changing pump modes or infusion rates) increased the likelihood of requesting a second nurse for double‐checking, which increased adherence (qualitative) (van Stralen et al. 2024).

##### 3.4.2.6 Calculation Difficulty and Confidence

Nurses reported that when medication calculations were particularly difficult, they were more inclined to perform a double‐check during medication administration, although the guidelines required calculation checks only during the preparation phase (qualitative) (van Stralen et al. 2024).

##### 3.4.2.7 Workload Pressure and Staffing Constraints

Adherence decreased under workload pressure and staffing constraints (cross‐sectional) (Alsulami et al. 2014a). It declined further as workload increased due to greater clinical complexity, short admission periods, staff pressures, time pressure, and frequent interruptions (semi‐structured group interview) (Schutijser et al. 2019). Nurses were also less likely to perform double‐checking during peak workload periods (qualitative) (Dickinson et al. 2010).

##### 3.4.2.8 Shift Timing and Time of Administration

Adherence was consistent across shifts for most nurses; however, some demonstrated higher adherence during night shifts due to fewer interruptions (cross‐sectional) (Alsulami et al. 2014a). Optional double‐checking was more likely for medications administered after 18:00 (observational) (Westbrook et al. 2021).

##### 3.4.2.9 Day Type (Weekdays vs. Weekends)

Adherence on weekdays versus weekends varied by study context. Nurses perceived no difference in adherence between weekdays and weekends (cross‐sectional) (Alsulami et al. 2014a). In contrast, higher observed adherence on weekends than weekdays was reported (observational) (Alsulami et al. 2014b), and higher odds of mandated double‐checking on weekends (OR = 1.50 (1.02–2.21)) (observational) (Westbrook et al. 2021).

#### 3.4.3 Technology and Tool Factors

##### 3.4.3.1 Medication Chart Format (Paper vs. Electronic Medication Management)

Nurses’ adherence to optional double‐checking was higher when paper‐based medication charts were used during administration compared with electronic medication management systems (observational) (Westbrook et al. 2021).

##### 3.4.3.2 EHR and Barcode Systems

Electronic health records (EHRs) with medication administration records and barcode medication administration systems supported double‐checking by enabling digital completion of five of the seven checking steps through automated comparison of scanned patient and medication data with the EHR and discrepancy alerts. These systems reduced the time required for double‐checking and were perceived as a facilitator of adherence (semi‐structured group interview) (Schutijser et al. 2019).

#### 3.4.4 Physical Environment Factors

##### 3.4.4.1 Interruptions During Administration

Interruptions in busy, noisy, and easily accessible medication rooms impaired nurses’ ability to perform double‐checking (qualitative) (Dickinson et al. 2010). Significantly higher adherence to double‐checking steps on weekends than on weekdays was attributed to fewer interruptions on weekends compared with weekdays (cross‐sectional) (Alsulami et al. 2014a).

##### 3.4.4.2 Overcrowding in Medication Areas and Wards

Overcrowding was identified as a barrier to double‐checking. Crowded medication rooms disrupted nurses’ ability to prepare medications and perform independent double‐checks, contributing to lower adherence (qualitative) (Dickinson et al. 2010). At the ward level, a busy ward environment made it harder to find a second nurse for double‐checking during medication administration, which reduced adherence (qualitative) (van Stralen et al. 2024). Collectively, overcrowding in both medication rooms and wards was associated with lower adherence.

##### 3.4.4.3 Clinical Emergent Situations

Adherence was found to be lower during emergent clinical situations (cross‐sectional) (Nakamura et al. 2021).

#### 3.4.5 Organizational Factors

##### 3.4.5.1 Availability of a Second Checker

Nurses reported that the unavailability of a second checker reduced adherence (cross‐sectional) (Alsulami et al. 2014a). Findings further showed that junior nurses sometimes struggled to find approachable or trustworthy colleagues as second checkers, which reduced adherence (qualitative) (Dickinson et al. 2010). Increased staffing (including trainees) facilitated double‐checking (semi‐structured group interview) (Schutijser et al. 2019). Similar evidence from transfusion bedside checking showed lower second‐person involvement for certain steps due to availability constraints (cross‐sectional) (Nakamura et al. 2021). Nurses were more likely to perform double‐checking when a colleague was immediately present, but often skipped it during busy periods or when no colleague was available (qualitative) (van Stralen et al. 2024). Overall, these studies consistently showed that second‐checker availability influenced adherence.

##### 3.4.5.2 Approachability and Trustworthiness of the Second Nurse

Adherence decreased when junior nurses hesitated to ask senior nurses for verification, often because they worried about annoying them or appearing to question their expertise (qualitative) (Dickinson et al. 2010).

##### 3.4.5.3 Ward/Unit Type (Orthopedics)

Adherence to mandated double‐checking was significantly higher on orthopedics wards than on other units (OR = 2.1; 95% CI: 1.08–4.11) (observational) (Westbrook et al. 2021).

##### 3.4.5.4 Cultural Norms of Double‐Checking

Adherence was significantly influenced by cultural norms, with some wards exhibiting established practices of omitting verification steps (focus group) (Gill et al. 2012). This culture manifested in widespread non‐adherence to patient ID verification and mandated medication double‐checks, with staff often rationalizing non‐adherence as protocol impracticality or substituting alternative verification methods (Gill et al. 2012).

##### 3.4.5.5 Safety Culture

Negative safety cultural norms, such as systematic omission of verification steps, were associated with lower adherence (focus group) (Gill et al. 2012). In contrast, adherence was higher in units where nurses perceived their team culture as collegial, critical, and open to feedback (semi‐structured group interview) (Schutijser et al. 2019).

##### 3.4.5.6 Access to Pediatric Medication Resources

Adherence decreased when nurses faced barriers to accessing pediatric medication resources needed to assist and guide medication administration, particularly for complex pediatric administrations (qualitative) (Dickinson et al. 2010).

##### 3.4.5.7 Alternative Means of Double‐Checking

Non‐adherence to patient identification verification within double‐checking was reported when nurses used informal alternatives (e.g., confirming identity with the patient or with the parent) (focus group) (Gill et al. 2012).

##### 3.4.5.8 Acceptability and Perceived Practicality of Double‐Checking

Nurses consciously deviated from double‐checking, citing impracticality, and admitted to deliberately omitting patient ID verification across units, including pediatric intensive care (focus group) (Gill et al. 2012). Such omissions often led to failure to verify correct medication chart usage (Gill et al. 2012).

##### 3.4.5.9 Preparation of Injectable Medications by the Hospital Pharmacy

Preparation of injectable medications by the hospital pharmacy facilitated the double‐checking process by reducing the time required for double‐checking, which may have increased adherence (semi‐structured group interview) (Schutijser et al. 2019).

##### 3.4.5.10 Limited Visibility of the Medication Preparation Process

Limited visibility into the hospital pharmacy's medication preparation process was a barrier to correctly performing double‐checking, which may have reduced adherence (semi‐structured group interview) (Schutijser et al. 2019).

#### 3.4.6 External Environmental Factors

The reviewed studies did not examine the impact of external environmental factors (e.g., regulatory policies, economic constraints, or social influences), which revealed a research gap.

### 3.5 Double‐Checking Adherence‐Improvement Strategies and Their Effectiveness

Table 6 summarizes the characteristics of the studies evaluating the strategies to improve nurses’ double‐checking adherence, with outcomes reported as process measures (documentation or observed verification) in quality improvement designs.

**TABLE 6:** Details of strategies examined to promote nurses’ adherence to double‐checking.

Author (year), location	Study duration and setting	Sample	Strategy components	Impact on adherence to double‐checking
Hospodar (2007), United States	Initiated Jan 2006; acute inpatient rehabilitation nursing units (two units; 31 beds and 29 beds). Pre vs. ∼4 weeks post implementation audit of diabetic flow sheets.	Insulin administrations audited via diabetic flow sheets: baseline 187 administrations; post 230 administrations.	Documentation‐focused workaround: half‐inch co‐signature stickers signed by the verifying nurse and placed on the diabetic flow sheet by the administering nurse; in‐depth staff education; posters explaining policy/process; “STICK TO IT” signage; stickers/instructions placed on medication refrigerator doors.	Documented double‐checks increased from 65/187 (35%) to 167/230 (72%) after the sticker process was introduced.
Raja Lope et al. (2009), Malaysia	Two 2‐week observation phases: Feb 2005 (baseline) and Feb 2006 (post). NICU setting (34 beds). Nurses were covertly observed for adherence to medication administration steps.	Medication doses observed: 188 baseline; 169 post. Also reported nurses observed: 50 (phase 1) and 51 (phase 2).	Remedial package after baseline: re‐education program (feedback + lectures + posters) plus implementation of a standard operating procedure for medication administration steps.	For the step “countercheck calculation of drugs”: non‐adherence worsened from 39/188 (20.7%) to 161/169 (95.3%). This corresponds to adherence dropping from 79.3% to 4.7%, attributed in the discussion to difficulty getting a second nurse (staffing/colleague burden).
Subramanyam et al. (2016), United States	Aug 2014–Feb 2015. Tertiary pediatric hospital's anesthesia radiology imaging locations.	Cases where infusion pumps were used for infusion medications; exact *n* not reported.	Two‐person verification system implemented via iterative PDSA testing. Frequent educational meetings; written reminders; visual reminders/displays (initial sticker idea abandoned; later visual reminders used); continuous feedback; run‐chart sharing to build buy‐in; workflow/IT integration by modifying EMR documentation and embedding the check in the procedural timeout.	Two‐person verification of infusion pump programming increased from 0% to 90% and was sustained; 4 programming errors were detected and corrected before administration; no meaningful delay in case starts reported (>90% no delay; 95% stakeholders reported no delay).
Fang et al. (2016), China	2011–2015 hospital‐wide medication management improvements. Standardized administration policy active since Jan 2013.	Hospital‐wide implementation; adherence assessed via on‐site inspections and retrospective review of the nursing record system.	Two licensed healthcare professionals must perform a standardized independent double‐check prior to barcode‐assisted administration of high‐alert meds. Technology support: barcode scanning and traceability, plus ongoing inspections/audits as part of the MMU program.	Reported 100% implementation rate of the independent double‐check policy since Jan 2013, based on on‐site inspection and retrospective nursing record review.

#### 3.5.1 In‐Depth Education, Poster, and Half‐Inch Sticker for Co‐Sign Diabetic Flow Sheet

One study implemented a multifaceted strategy to improve documentation adherence to policy‐mandated double‐checks for insulin administration in an acute inpatient rehabilitation facility (Hospodar 2007). The strategy combined in‐depth staff education, posters/signage outlining the policy, and a co‐signature system using half‐inch stickers on diabetic flow sheets. After verifying the insulin dose, the checking nurse signed the sticker, which the administering nurse then affixed to the patient's flow sheet (containing dedicated fields for administration time and blood glucose level). Stickers and instructions were placed on medication refrigerator doors, while “Stick to It” signs were displayed on medication room doors to promote visibility and engagement. Over approximately four weeks, this strategy increased documentation adherence from 35% (65/187) to 72% (167/230).

#### 3.5.2 Re‐Education Program and Standard Operating Procedure Implementation

The effects of a re‐education program and standard operating procedures on medication administration practices in a neonatal intensive care unit were evaluated using covert direct observation of nurses (Raja Lope et al. 2009). The observations spanned two 2‑week phases: baseline and follow‐up. The re‐education program comprised feedback sessions, lectures, and educational posters. Notably, adherence to the double‐checking step “countercheck calculation of drugs” decreased significantly from 79.3% to 4.7% (with non‐adherence rising from 20.7% [39/188] to 95.3% [161/169]). This decline was attributed primarily to the unavailability of a second nurse and the reluctance to interrupt colleagues already engaged in other duties. Several other administration steps showed improvement post‐intervention, while the “witness for drug administration” step remained rare in both phases (∼95% non‐adherence).

#### 3.5.3 Educational Meetings, Reminders, Feedback, and Sharing of Knowledge

A quality improvement initiative at a U.S. tertiary pediatric hospital's anesthesia radiology imaging service enhanced safety through 24 plan–do–study–act (PDSA) cycles (Subramanyam et al. 2016). The strategy included: (1) educational meetings and job aids emphasizing safety checks, standardized infusion pump programming, role clarification, and a data collection form to track double‐checking; (2) written reminders in work areas; (3) visual displays/reminders at high‐risk failure points (an initial sticker approach was trialed but discontinued due to resistance and space issues); (4) ongoing feedback via weekly updated run charts, a key driver diagram, and regular reviews; and (5) knowledge sharing on run‐chart performance and intercepted errors, reinforced by EMR documentation updates and procedural timeout integration. Adherence to double‐checking (two‐person verification of infusion pump programming) rose from 0% to 90% and was sustained, with four programming errors caught and corrected before administration, without notable delays in case start.

#### 3.5.4 Double‐Checking Protocol, Barcode Tracking, and Regular Audits

In a four‐year medication management and safety improvement program (2011–2015) at a 3200‐bed academic medical center in China, the hospital required, from January 2013, a standardized independent double‐check by two licensed healthcare professionals before barcode‐assisted medication administration of narcotics and other high‐alert medications, including insulin infusion, chemotherapeutics, and intravenous heparin. Narcotic labels included a barcode and administration information, and barcode scanning, used in conjunction with an online inquiry system, enabled traceability across the medication‐use process from prescribing to administration. Adherence with the double‐check requirement was assessed through on‐site inspections and retrospective review of the nursing record system, which showed a 100% implementation rate from January 2013 onward (Fang et al. 2016).

## 4 Discussion

### 4.1 Implications for Nursing Practice

#### 4.1.1 Ensuring True Independence in Double‐Checking

Nurses should perform double‐checking as truly independent verifications, rather than as confirmation of a colleague's check. Observational data show that even when adherence to double‐checking exceeds 90%, only about 1% of checks are fully independent (Westbrook et al. 2021). Most checks are primed, whereby the first nurse's information or behavior influences the second nurse's review, and these yielded no significant benefit in reducing medication errors (Westbrook et al. 2021). Non‐independent checks, therefore, tend to confirm the first nurse's expectations and may fail to detect errors (Konwinski et al. 2024).

To minimize priming, each nurse involved in double‐checking should verify all the required details independently and without shared expectations. After the first nurse prepares a dose, the second nurse should check the dose directly from the original order and compare it with the prepared medication. The second nurse should not review or repeat the first nurse's verification until after completing their own independent check. Guidelines from the Institute for Safe Medication Practices emphasize that independent double‐checking requires the second checker to verify without cues from the first (Institute for Safe Medication Practices 2013), as two checkers working independently are unlikely to make the same mistake (Institute for Safe Medication Practices 2013). Research supports this principle, showing that independent double‐checking detected more dosing errors than checks influenced by the first nurse's input (Koyama et al. 2020). Training programs and reminders should therefore reinforce independent double‐checking and discourage disclosure of expected results before verification is complete.

#### 4.1.2 Optimizing the Work Environment and Workflow

Nurses are more likely to follow double‐checking procedures when interruptions and time pressures are minimized (Schroers et al. 2025). Units should establish “quiet zones” for medication preparation and double‐checking to reduce distractions (Kellett et al. 2024; Owen et al. 2023). Visible reminders and checklists placed at medication stations can be useful, such as by prompting staff to complete each step (Chen et al. 2023). Assigning nurses in buddy pairs or with overlapping shifts can ensure a second checker is readily available, preventing delays that tempt nurses to skip the process. High workload and chaotic ward layouts have been identified as barriers to proper double‐checks. Nursing teams can reorganize workflows and layouts so that the first nurse can access a second nurse without leaving medication administration tasks unfinished. Units can also standardize the timing of double‐checking during medication rounds to support timely completion. Incorporating technology is also a viable consideration. Barcode scanning, smart infusion pumps, and electronic co‐signature requirements can prompt double‐checking and support documentation (Mulac et al. 2021). However, technology must be user‐friendly and well integrated into the workflow (Grailey et al. 2024; Johnson et al. 2008; Karsh et al. 2011). If electronic systems are slow or difficult to use, they can lead to technology abandonment (in voluntary use contexts) or increase time demands and encourage workarounds (in mandatory use contexts).

### 4.2 Implications for Nursing Policy

#### 4.2.1 Risk‐Based and Well‐Defined Policy

Healthcare policymakers and nursing management should align double‐checking policies with evidence and risk levels (Koyama et al. 2020). Policies should emphasize when double‐checking is required, particularly for high‐alert medications or pediatric dose preparations (Westbrook et al. 2021). This risk‐based approach prioritizes high‐risk medications, which can reduce the likelihood of these medications being missed (Konwinski et al. 2024). Any mandated double‐checking procedure must define independent double‐checking and specify steps required for adherence monitoring and evaluation. Guidelines should require independent calculations, independent documentation, and independent patient identity verification (Schwappach et al. 2016). Management should regularly review incident reports and current evidence to update double‐checking policies. When evidence shows that a double‐checking practice is ineffective, policies should be revisited. When evidence shows benefits in specific contexts (e.g., chemotherapy administration), policies should retain double‐checking and monitor adherence. Clear, evidence‐based standards can support adherence by clarifying the rationale for double‐checking and the actions required for independence (van Stralen et al. 2024). Policymakers can incorporate these standards into accreditation and licensing requirements to reinforce independent double‐checking as a component of quality care. Risk‐based and well‐defined policies can position double‐checking as a targeted safety practice rather than a routine obligation.

#### 4.2.2 Adequate Staffing and Resources

Ensuring adequate staffing and resource availability is a key nursing management responsibility for maintaining patient safety and reducing rationing or missed care (Nantsupawat et al. 2022; Uchmanowicz et al. 2024). Because medication double‐checking requires a second nurse and additional time, insufficient staffing and time pressure can reduce adherence in practice (Hewitt et al. 2016; Westbrook et al. 2021). Chronic understaffing can force nurses to skip safety steps (e.g., double‐checking) (van Stralen et al. 2024). Nursing leadership should advocate for staffing levels that account for the time needed for mandated safety procedures (van Stralen et al. 2024), such as adjusting nurse‐to‐patient ratios or assigning extra nurses during peak medication administration periods to serve as second checkers (Han et al. 2021). Policymakers at institutional and system levels should recognize that unfunded safety mandates are unsustainable (Hewitt et al. 2016). Thus, if regulations or policies mandate double‐checking, budgets and schedules should allocate the required nursing hours (Subramanyam et al. 2016). Practical strategies include dedicated medication safety nurses, overlapping shifts during high‐volume periods, or electronic coordination systems and real‐time remote double‐checking technologies to reduce time locating a second nurse.

#### 4.2.3 Integration of Double‐Checking Into Workflow and Technology

Managers and healthcare leaders should integrate double‐checking into workflows in a standardized, supportive way (Borrelli et al. 2025; McComas et al. 2014; Westbrook et al. 2021). One approach is embedding double‐check prompts and documentation into EHRs or medication administration systems (Koyama et al. 2020). For instance, systems can require a second nurse's login or confirmation before administering high‐risk medications (Kinlay et al. 2021). Such integration incorporates adherence into routine documentation and reduces reliance on individual reminders. However, the system must be designed to ensure truly independent checks, for example, by allowing each nurse to enter calculations independently and flagging discrepancies for resolution (Powers et al. 2018). Management should consult nurses during the implementation of new technologies or processes to prevent unintended burdens (van Houwelingen et al. 2024). If forms or EHR pop‐ups are too time‐consuming or confusing, staff may adopt workarounds that defeat the purpose (Blijleven et al. 2017). Continuous quality improvement is essential to monitor implementation, eliminate unnecessary steps, and maintain independence and accuracy (Kinlay et al. 2021). Hospitals could adopt standardized checklists or forms to guide both checkers through verification steps (White et al. 2010). Managers should also provide designated quiet zones for medication preparation to minimize interruptions (Huckels‐Baumgart et al. 2021; Westbrook et al. 2021). These system‐level interventions enhance safety by decreasing dependence on individual vigilance under time pressure.

#### 4.2.4 Fostering a Safety Culture and Accountability at the Organizational Level

Work and safety cultures vary across settings (Lin et al. 2025); therefore, policymakers and senior management should promote a safety culture that consistently expects and reinforces double‐checking where feasible and relevant (Schwappach et al. 2016). Within a just culture, accountability for both staff and management should be clear, while responses to errors should prioritize learning, open reporting, and system improvement rather than blame (Rogers et al. 2017). A just culture encourages staff to report near‐misses and errors detected during double‐checking, which can facilitate organizational learning (Boysen 2013). Also, adherence can be monitored through audits and feedback at the unit level (Ivers et al. 2012), and root cause analysis should address infrastructure, process, and outcome barriers when compliance is low (Or et al. 2014). When leadership clearly communicates that patient safety takes precedence over speed, it may strengthen a safety‐first culture and support sustained adherence to double‐checking.

### 4.3 Future Research

#### 4.3.1 Standardizing Definitions and Measurement of Adherence

This review observed inconsistencies in how prior studies define double‐checking and measure adherence. To improve the knowledge, future research should establish clear definitions that distinguish independent double‐check from double‐signature or a primed check. Standardized metrics for assessing adherence in practice are also essential. Many organizations’ double‐checking policies lack explicit definitions and are applied inconsistently, which undermines effectiveness (Fossum et al. 2016; Schutijser et al. 2019; Subramanyam et al. 2016). Few studies reported whether observed double‐checks were truly independent or influenced by the first nurse (Dickinson et al. 2010; Pfeiffer et al. 2020). A 2020 systematic review found that most studies failed to distinguish independent from primed double‐checking (Koyama et al. 2020), with only two describing their process as independent, one reporting counts of independent versus primed checks, and none analyzing error rates separately by check type (Koyama et al. 2020). These gaps limit cross‐study comparisons and meta‐analysis. Future studies should define an independent double‐check as a process in which the first nurse shares no checking outcomes or expectations with the second nurse, who then independently verifies all details against the original order. Adherence should be assessed via direct observation or structured self‐report documenting completion of predefined steps, such as independent dose calculation and patient identity verification. A shared, standardized checklist would promote consistent reporting and facilitate data pooling across sites.

#### 4.3.2 Exploring Contextual and System‐Level Influences

Further research is needed to understand how contextual and system factors affect nurses’ adherence to double‐checking, which can inform system‐level improvements. Factors such as unit culture, hospital policies, workload, and regulatory environments shape adherence (Ek et al. 2022). Different settings, such as intensive care units, oncology wards, and general wards, face unique challenges and solutions (Schutijser et al. 2019). Ethnographic and qualitative studies can reveal how and why clinicians do their work (Or 2015; Or et al. 2011, 2014; Schutijser et al. 2019; van Stralen et al. 2024). External factors, including regulatory mandates and litigation fears, likely impact behavior but are understudied. Comparing institutions with strict versus flexible policies can identify system features that promote or hinder adherence. Future work should also explore how physicians, pharmacists, and administrators influence nursing double‐checking. Human factors engineering approaches can map interactions between the physical environment, task usability, and team communication to facilitate behaviors (Karsh et al. 2006; Konwinski et al. 2024).

#### 4.3.3 Rigorous Evaluation of Improvement Strategies

The current evidence base lacks high‐quality trials on double‐checking and related strategies, which limits the ability to draw causal inferences about their effectiveness (Douglass et al. 2018; Hewitt et al. 2016; Koyama et al. 2020). Future research should focus on well‐designed experiments. For example, researchers should compare mandatory independent double‐checking with single‐checking to measure error rates in high‐risk medications. Multi‐site studies can help assess whether findings are applicable across different settings. Because complex strategies often include education, reminders, and technology, studies should evaluate each component separately to identify the most effective elements. Conducting process evaluations alongside outcome studies can help clarify how behavior change occurs and determine which elements promote independent double‐checking.

#### 4.3.4 Long‐Term Outcomes, Clinical Impact, and Economic Considerations

Future research should assess the long‐term sustainability, clinical impact, and cost‐effectiveness of double‐checking practices, as this evidence will inform resource allocation toward effective medication safety strategies. First, most studies focus on immediate outcomes, with limited evidence on long‐term adherence (Ament et al. 2015; Liu et al. 2022), so longitudinal research is needed to determine sustainability. Second, the direct link between enhanced double‐checking and reduced patient harm remains unclear due to challenges in establishing causality (Konwinski et al. 2024; Koyama et al. 2020). Larger or simulation studies could better estimate errors prevented by high‐quality double‐checking. Third, economic evaluations are crucial for guiding policy, as double‐checking requires nursing time and resources (Westbrook et al. 2021). Future work should quantify the cost–benefit ratio by comparing savings from avoided adverse events with additional staffing and resource costs (Elliott et al. 2021; Hodkinson et al. 2020). Modeling and cost‐effectiveness analyses can identify conditions where double‐checking offers net benefits and evaluate alternatives like barcode systems (Jessurun et al. 2022).

## 5 Conclusions

This systematic review confirms that nurses’ adherence to medication double‐checking is a complex, system‐level issue rather than solely a matter of individual diligence and that it varies across settings. Key barriers, such as the absence of a second nurse, frequent interruptions, a poor clinical environment, insufficient nurse experience, low perceived risk or necessity of double‐checking, and high workload or time pressure, highlight the complexity of systemic problems that are difficult to address through isolated interventions. Consistent with the “system thinking” perspective, deviations and non‐adherence often originate from system problems rather than only individual shortcomings.

Few studies have evaluated strategies to improve double‐checking, but those that do often report better outcomes with multicomponent approaches, such as education, reminders, and technology. However, these can fail if systemic issues like staffing shortages remain unaddressed; for instance, adherence declined when no second nurse was available despite a new protocol.

Improving adherence requires coordinated efforts at both practice and organizational levels. Frontline strategies include creating quiet zones to minimize distractions, using checklists or visual cues, and fostering a culture where nurses feel empowered to double‐check medications independently. Organizationally, investing in adequate staffing, resources, and leadership commitment is essential to support proper double‐checking and cultivate a safety‐focused culture.

From a research perspective, high‐quality studies are needed to identify effective interventions, develop standardized measures of adherence, and understand how contextual factors influence compliance. Future research should also evaluate whether improved double‐checking reduces medication errors and patient harm, and assess the long‐term sustainability and cost‐effectiveness of these strategies. Ultimately, embedding double‐checking into routine workflows and organizational culture without overburdening staff is crucial for achieving and maintaining high adherence and enhancing medication safety.

## Author Contributions

Conceptualization: Calvin K. L. Or, Junyu Zhao. Protocol development: Calvin K. L. Or, Junyu Zhao. Search strategy development: Calvin K. L. Or, Junyu Zhao, Yigong Peng. Literature search execution: Junyu Zhao. Screening and study selection: Junyu Zhao, Yigong Peng; discrepancies resolved by Calvin K. L. Or. Data extraction: Junyu Zhao, Yigong Peng; validation: Ao Shan, Hao Liu. Risk of bias/quality assessment: Junyu Zhao, Yigong Peng. Data synthesis and interpretation: Hao Liu, Ao Shan, Junyu Zhao, Calvin K. L. Or. Manuscript drafting: Junyu Zhao, Hao Liu, Ao Shan, Calvin K. L. Or. Critical revisions for important intellectual content: Hao Liu, Ao Shan, Junyu Zhao, Calvin K. L. Or. Supervision and administration: Calvin K. L. Or.

## Funding

The authors have nothing to report.

## Ethics Statement

This study was a systematic review of published literature and did not involve recruitment of human participants, intervention/interaction with participants, or access to identifiable individual‐level data. Therefore, ethics committee approval (including the committee name and approval number) and informed consent (written or verbal) were not required.

## Conflicts of Interest

The authors declare that they have no known competing financial interests or personal relationships that could have appeared to influence the work reported in this paper.

## Data Availability

The data that support the findings of this study are available from the corresponding author upon reasonable request. Template data collection/extraction forms are not publicly available. All data extracted from the included studies, and all data used for the analyses, are fully reported in the manuscript (Results, Tables 4 and 5, and the accompanying narrative synthesis); therefore, no separate data set has been deposited. No analytic code is available because no quantitative meta‐analysis was conducted, and no custom code was generated. No other materials are publicly available.

## Specific search term in literature search

### 1

Search terms: “nurs* AND (“double check” OR “double checking” OR “double‐check” OR “double‐checking” OR ((check* OR verif* OR confirm* OR administer* OR dispens*) AND (“two nurses” OR “two people” OR “two person” OR “second person” OR “second nurse”))).”
